# Understanding Acceleration-Based Load Metrics: From Concepts to Implementation

**DOI:** 10.3390/s25092764

**Published:** 2025-04-27

**Authors:** João Freitas, Alexandre Moreira, João Carvalho, Filipe Conceição, Luisa Estriga

**Affiliations:** 1 Faculty of Sport, University of Porto, 4200-450 Porto, Portugal; up201706448@edu.fade.up.pt (J.F.); armoreira@sporting.pt (A.M.); lestriga@fade.up.pt (L.E.); 2 COD, Centre of Sports Optimization, Sporting Clube de Portugal, 1600-464 Lisbon, Portugal; 3 CEIIA, Engineering and Product Development Center, 4450-017 Matosinhos, Portugal; joao.mcarvalho@ceiia.com; 4 Algoritmi—Embedded Systems Research Group, Universidade do Minho, 4804-533 Guimarães, Portugal; 5 CIFI2D, Centre for Research, Education, Innovation and Intervention in Sport, University of Porto, 4200-450 Porto, Portugal; 6 LABIOMEP, Porto Biomechanics Laboratory, University of Porto, 4200-450 Porto, Portugal

**Keywords:** Jerk, Dynamic Stress Load, accelerometry, workload, Accel’Rate, body load

## Abstract

Accelerometer-based wearables offer a cost-effective solution for managing match and training loads in invasion team sports. However, a multitude of acceleration-derived metrics, each employing different algorithms, has led to inconsistent and incomparable outcomes across studies and devices. This article reviews the mathematical procedures underlying whole-body mechanical load metrics, clarifies their conceptual differences, and proposes refinements to enhance standardization. Synthetic data were employed to investigate conceptual differences, while experimental accelerometric data (463 time series) from a set of elite handball training sessions (involving 16 players) were used to implement the corrected equations and analyze statistical relationships. Analysis of synthetic data revealed that derivative-based metrics, such as Jerk Modulus (typically referred to as Player Load) and corrected Accel’Rate (cAccel’Rate), tend to amplify noise compared to acceleration-based metrics, such as universal Dynamic Stress Load (uDSL) and Body Load. Experimental results indicated that when metrics were summed, their values were nearly identical. In time-series comparisons, Jerk Modulus and cAccel’Rate were predictably found to be nearly identical, while Body Load was the most distinct. Acceleration-based metrics are preferable to derivative-based ones. Sports scientists should lead the design and validation of such metrics, ensuring methodological rigor, transparency, and innovation while preventing commercial interests from introducing rebranded variables with undisclosed scaling factors and unclear calculations.

## 1. Introduction

The revolution arising from the availability of microcontrollers and MEMS (microelectromechanical systems), along with their advantages of being small, inexpensive, and easy to use, has introduced accelerometry as a promising methodology and tool for measuring physical activity and exertion in elite sports [1]. However, this has been an exciting yet challenging journey, as it remains unclear how acceleration load can be effectively used to quantify activity intensity and the volume of a team sports training session—both key parameters for training control [2]. Additionally, there has been ongoing debate about the ‘training load’ construct, its multidimensional nature [3]—linking external load, internal load, and contextual factors—and whether the term is being properly used in the field of sports science [2,4]. While some researchers question the use of the term ‘training load’ [2], others [5] argue that it has undergone extensive development and is now widely accepted in the field.

For decades, sports scientists have been using accelerometers to quantify mechanical load, initially by counting the number of instances in which acceleration exceeded a threshold [6,7,8,9]. Similarly, the number of acceleration and deceleration events [10] began to be explored in elite team sports to monitor physical demands in training and competition, along with more complex loading metrics, initially referred to as accumulated load [11].

Many of these metrics have been developed by commercial companies [1,2,10,12] to provide objective information to both practitioners and the sports science community, while also seeking scientific recognition. However, when reported by authors, crucial parameters of some of these metrics have been shown to be misinterpreted, overlooked, or purposely vague, which can compromise their universal applicability [4]. The multitude of such metrics is even more problematic when two distinct commercial systems claim to use the same metric but apply different or undisclosed sample rates, filters, thresholds, scaling factors, fudge factors, etc., rendering comparisons hopeless [12,13].

Other research has compared various external load metrics, finding differences in absolute values, mainly due to the scaling factors in each metric [4,14]. However, unique metrics like Body Load [12], Dynamic Stress Load (DSL) [15], and Accel’Rate [16] have not yet been explored. Additionally, these studies did not attempt to rectify the equations to improve standardization; they merely used the already published metrics and noted their statistical similarities.

Beyond kinematic data (accelerometric and positional), coaches have access to various other sources of information that can assist in training and match control. With the rise of artificial intelligence (AI), speculation has increased about its potential to open new avenues for extracting more reliable information [17,18], particularly to enhance load monitoring and different system responses related to adaptation, fatigue, and recovery [19].

However, the output quality of these models is highly dependent on the input data’s quality. The colloquial term “garbage in, garbage out” has been coined by researchers to highlight the impact of poor-quality input on the output [20]. Thus, while advances in wearable technology and AI may help refine training load monitoring, they also introduce new challenges regarding data quality and interpretation.

In this methodological article, we specifically aim to review and discuss the mathematical procedures underlying whole-body mechanical load metrics, clarify their conceptual differences, and propose refinements to enhance standardization and comparability across different hardware devices. Additionally, implementing the corrected formulas using real-world accelerometric data allows us to explore and compare the metrics, providing a clearer understanding of their relationships and distinctiveness.

Therefore, our goal is not to introduce new acceleration-based exertion metrics, but rather to revisit some of the most commonly used ones and introduce minimal corrections that preserve their fundamentals while enabling their use across different hardware, thereby enhancing their value.

## 2. Analysis of External Load Metrics Based on Acceleration

Most of the sports literature considers the acceleration as a vector in Cartesian coordinates, which we will denote as $\vec{a}=(ax,\;ay,\;az)$. This is typically assumed to be sampled at a regular rate, with a period that we will designate as ∆t. Thus, we refer to

$${\vec{a}}_{i}={\left(ax,\;ay,\;az\right)}_{i}=({ax}_{i},\;{ay}_{i},\;{az}_{i})$$

as the acceleration at the instant i·∆t.

Player Load is perhaps the most widely used metric in sports science for quantifying mechanical load through IMUs [1,2]. Several variations have already been used (primarily in the scaling factor) [14], but the equation first appearing in [11,21] is

$$PL_{i}=\sqrt{\frac{{\left({ax}_{i+1}-{ax}_{i}\right)}^{2}+{\left({ay}_{i+1}-{ay}_{i}\right)}^{2}+{\left({az}_{i+1}-{az}_{i}\right)}^{2}}{10^{2}}}$$

This definition is a temporally discretized equation reporting a quantity proportional to $\left\|\frac{d\vec{a}}{dt}\right\|$.

**Figure 1:**
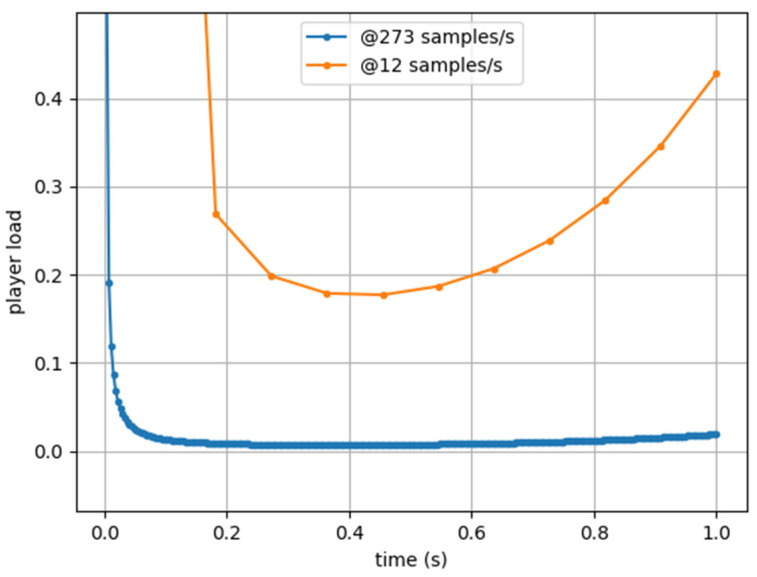
Player Load estimation using published equation [11,21] from artificial acceleration data sampled at 273 Hz (blue) and 12 Hz (orange).

The published version [11,21] presents two major issues: it includes an arbitrary numerical factor that obscures ∆t and prevents its modification, and it is highly sensitive to data noise. As a result, measurements of the same event taken at different sampling rates become incomparable, as illustrated in Figure 1. What the authors likely omitted, and what has since led to misunderstandings, is that the division by 10^2^ in the originally published equation is most likely intended as a scaling factor, but may in fact be a residual of the sampling rate.

Thus, it is advantageous to return to the mechanical foundation and define a corrected Player Load metric called the Jerk Modulus, as jerk represents the rate of change of acceleration over time, where the sampling rate is accounted for:

(1) $${Jerk\;Modulus}_{i}=\frac{1}{∆t}\sqrt{{\left({ax}_{i+1}-{ax}_{i}\right)}^{2}+{\left({ay}_{i+1}-{ay}_{i}\right)}^{2}+{\left({az}_{i+1}-{az}_{i}\right)}^{2}}$$

**Figure 2:**
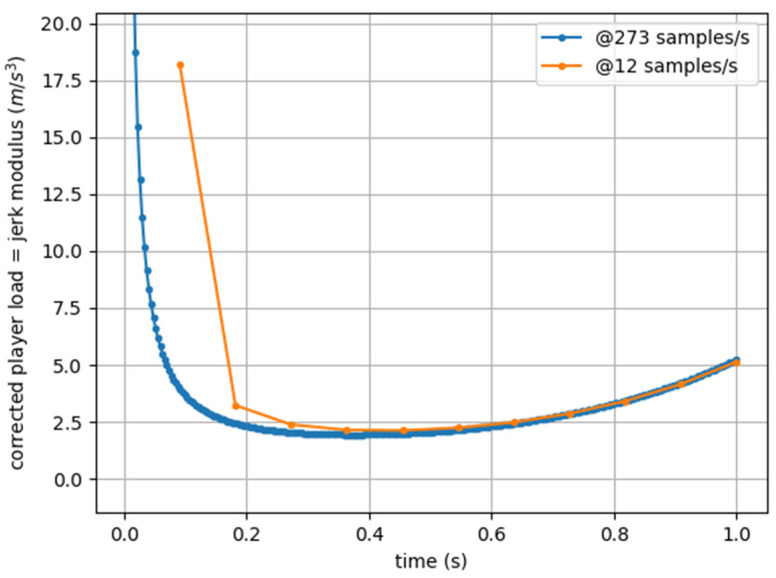
Jerk Modulus (corrected Player Load) computed from artificial acceleration data sampled at 273 Hz (blue) and 12 Hz (orange).

Now, except for a few initial points (which are different because the proposed discretization is progressive and not centered), the values are essentially identical (with the curves being overlapped) and comparable (see Figure 2).

**Figure 3:**
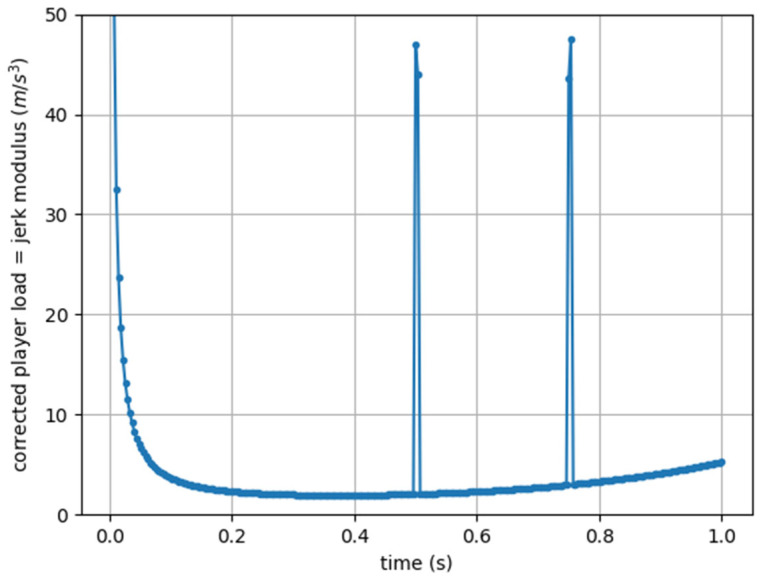
Jerk Modulus (corrected Player Load) computed from artificial acceleration data sampled at 273 Hz, with two points of disturbance (a value of 18 milliG).

The second disadvantage of the originally published Player Load formula is related to the fact that the derivative of acceleration is highly sensitive to noise. In the example shown in Figure 3, a point disturbance was deliberately introduced at two moments, with a value of 18 milliG (1% of the unperturbed value), and the effect is visible.

Other authors [16] have proposed a metric very similar to Player Load, referred to as Accel’Rate (i.e., acceleration rate), which measures the instantaneous changes in the magnitude of the 3-axis acceleration vector,

$$Accel’{Rate}_{i}=\left|\sqrt{{\left(a_{x_{i}}\right)}^{2}+{\left(a_{y_{i}}\right)}^{2}+{\left(a_{z_{i}}\right)}^{2}}-\sqrt{{\left(a_{x_{i-1}}\right)}^{2}+{\left(a_{y_{i-1}}\right)}^{2}+{\left(a_{z_{i-1}}\right)}^{2}}\right|.$$

This is equation can be compacted to:

$$Accel’{Rate}_{i}=\left|\;\left\|{\vec{a}}_{i}\right\|-\left\|{\vec{a}}_{i-1}\right\|\right|$$

**Figure 4:**
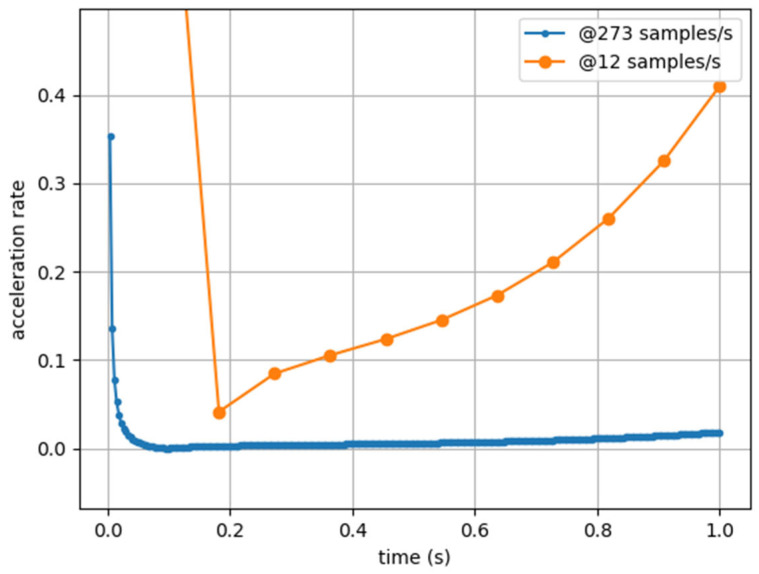
Accel’Rate computed from artificial acceleration data sampled at 273 Hz (blue) and 12 Hz (orange).

It is now clear that this metric is simply the temporally discretized version of the absolute value of the derivative of the acceleration modulus, i.e., $\left|\frac{d\left\|\vec{a}\right\|}{dt}\right|$, where the sampling rate was simply disregarded. Naturally, the impact of overlooking this factor has the same consequence as in the case of Player Load, i.e., the same activity or event, measured using two similar sensors placed at the same location (e.g., upper back) but operating at different sampling rates, will report incomparable Accel’Rate results, as illustrated in Figure 4.

Hence, it is useful to correct the definition of this metric by integrating the missing sampling rate factor, and to refer to the revised version as cAccel’Rate:

(2) $${cAccel’Rate}_{i}=\frac{1}{∆t}\left|\sqrt{{ax}_{i+1}^{2}+{ay}_{i+1}^{2}+{az}_{i+1}^{2}}-\sqrt{{ax}_{i}^{2}+{ay}_{i}^{2}+{az}_{i}^{2}}\right|$$

**Figure 5:**
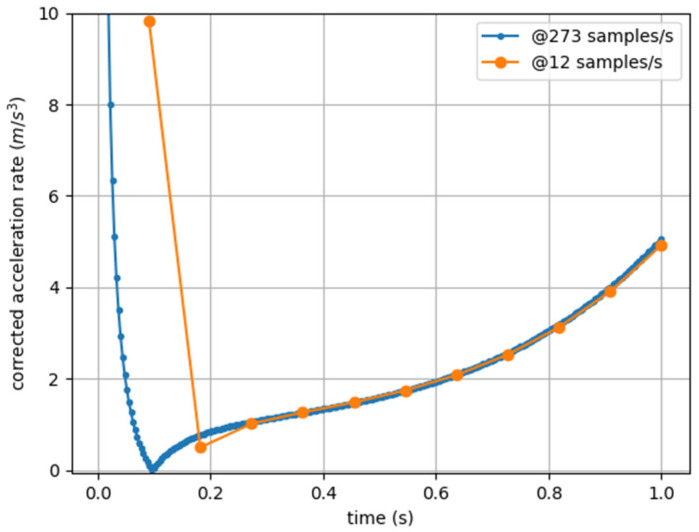
cAccel’Rate computed from artificial acceleration data sampled at 273 Hz (blue) and 12 Hz (orange).

As in the previous metrics analysis, with this correction, measurements taken at different acquisition rates become comparable, except for a few initial points (see Figure 5).

**Figure 6:**
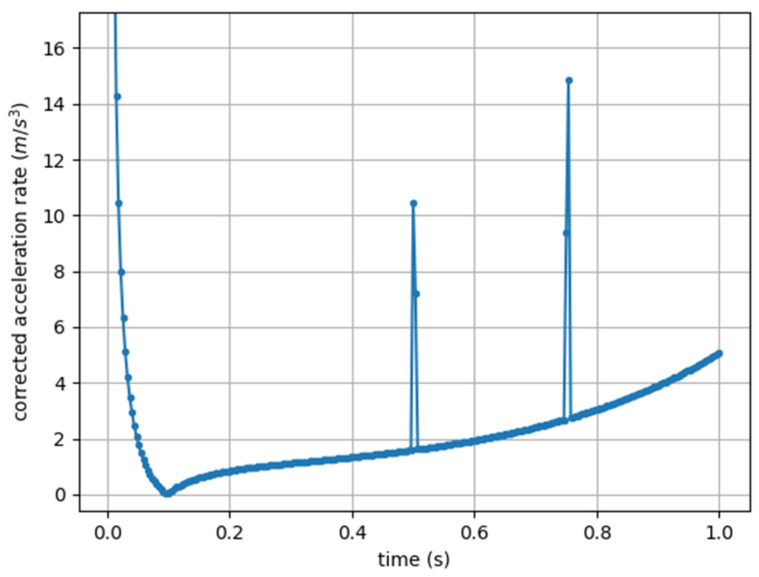
cAccel’Rate computed from artificial acceleration data sampled at 273 Hz, with two points of disturbance (a value of 18 milliG).

Similarly to the Jerk Modulus, the cAccel’Rate definition, being a derivative, is a quantity that is highly sensitive to noise. In Figure 6, the cAccel’Rate is shown for acceleration data containing two disturbed points, as previously shown.

Some external mechanical load metrics use acceleration instead of its derivative, following the principle that kinematic load increases supra-linearly with acceleration. Unlike derivative-based measures, these metrics must account for gravitational acceleration, which is approximately 10 m/s^2^ at rest but difficult to pinpoint due to the arbitrary orientation of both accelerometers and subjects. Therefore, these metrics include mechanisms to ignore gravitational acceleration and filter out minor efforts like slow walking.

An example of these metrics directly derived from acceleration is Body Load [12]:

$$bl\text{\_}{impact}_{i}=\left\{\begin{array}{c} \left\|{\vec{a}}_{i}\right\|-1⇐\left\|{\vec{a}}_{i}\right\|>1.25G\\ 0\;\;else \end{array}\right.$$

(3) $${BL}_{i}={bl\text{\_}impact}_{i}+{bl\text{\_}impact}_{i}^{3}$$

**Figure 7:**
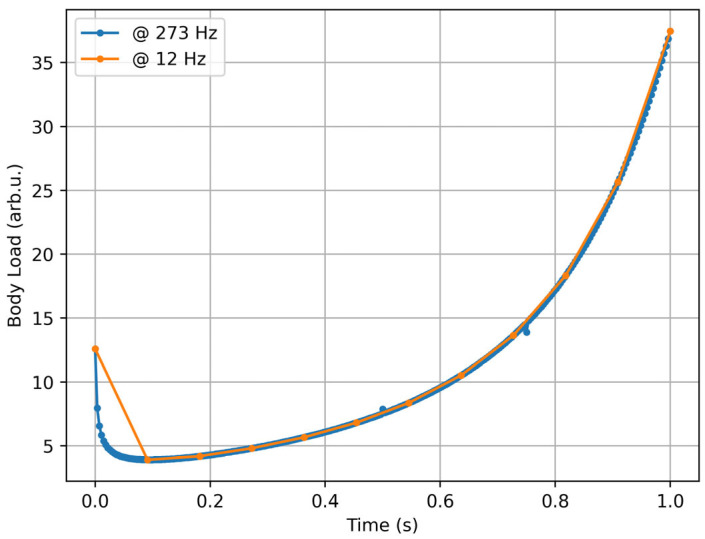
Body load [12] computed from artificial acceleration data sampled at 273 Hz (blue) and 12 Hz (orange), with two points of disturbance (a value of 18 milliG).

The concept of *bl_impact* aims to address the problem of gravitational acceleration, although it introduces a cut-off. The metric includes a linear term, which becomes relevant at low accelerations, and a cubic term, both of which preserve the orientation of the acceleration vector. Nonetheless, the metric is independent of acquisition rates and highly resistant to noise (see Figure 7).

Finally, Gaudino et al. [15] and others [22] report a metric called Dynamic Stress Load, without ever defining it precisely. It appears to be:

$$dsl\text{\_}{impact}_{i}=\left\{\begin{array}{c} \underset{0.1\mathrm{s window}}{\max}\left\|\vec{a}\right\|⇐\underset{0.1\mathrm{s window}}{\max}\left\|\vec{a}\right\|>2G\\ 0\;\;else \end{array}\right.$$

$${DSL}_{i}=\alpha \cdot {dsl\text{\_}impact}_{i}^{k}$$

The confusing indexes above appear to define the impact as the maximum sampled acceleration modulus over a (non-overlapping? moving?) 0.1 s window containing the sample *i* if it exceeds two times the gravitational acceleration (2G).

**Figure 8:**
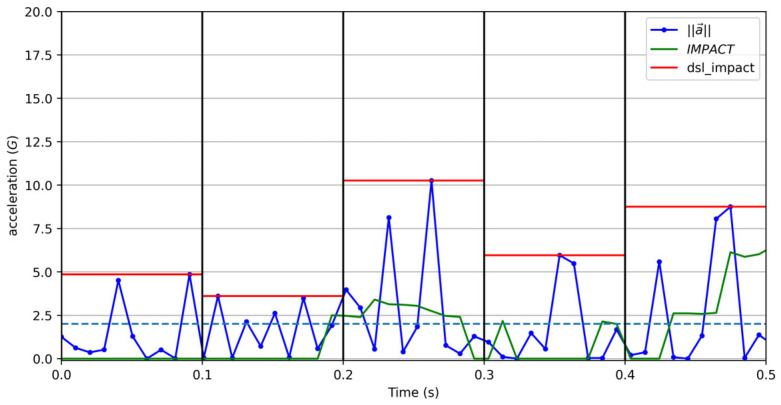
Comparison between the possible definition of impact in DSL by Gaudino et al. [15] (red) and the definition proposed in this study (green), computed from artificial acceleration data. The blue dashed line marks the 2 *G* threshold defined as the minimum acceleration value to be considered.

We tested multiple definitions and found no relevant differences among them. We prefer using a moving average instead of a maximum function, as it is significantly less sensitive to noise (see Figure 8). Thus, we define:

$${\tilde{y}}_{i}=\frac{10}{sample\;rate}\sum_{k=\left(-50\;ms\right)}^{\left(50\;ms\right)}\left|\left|{\vec{a}}_{i+k}\right|\right|$$

This procedure might demand an interpolation if the sample time interval is not a factor of 50 ms, or if it is not constant. Moreover, if the sample time interval is higher than 100 ms (i.e., sampling rate < 10 Hz), the moving average described above cannot be used; in such cases, calculating ${\tilde{y}}_{i}$ *=* $\left|\left|{\vec{a}}_{i}\right|\right|$ can serve as an option, regardless of the filtering process applied. With ${\tilde{y}}_{i}$ defined, we can define an impact as:

$$IMPACT_{i}=\left\{\begin{array}{c} {\tilde{y}}_{i},\;\;{\tilde{y}}_{i}>2\;G\\ 0,\;\;else \end{array}\right.$$

The impact definition proposed uses *G* as the acceleration unit, which is common within the tracking companies’ proprietary software. The *k* weighting factor associates the DSL measurements with biomechanical effects, approximated as a cubic function in Gaudino et al. [15]. The authors justify this by stating that “an impact of 4 *G* is more than twice as hard on the body as an impact of 2 *G*” [15], p. 861. Our own tests suggest that the *k* factor used by the authors is around 2.7, but this makes very little difference. In the absence of a clear value, and in favor of standardization, we adopted the value declared by Gaudino et al., *k* = 3.

The scaling factor (*α*) in Gaudino et al. [15] is not disclosed, but we recommend it to be the unity, as in all other cases above. Since this may not correspond exactly the original DSL, we refer to the revised version as the universal Dynamic Stress Load (uDSL), with *G* as the acceleration unit:

(4) $$uDSL_{i}=IMPACT_{i}^{3}$$

**Figure 9:**
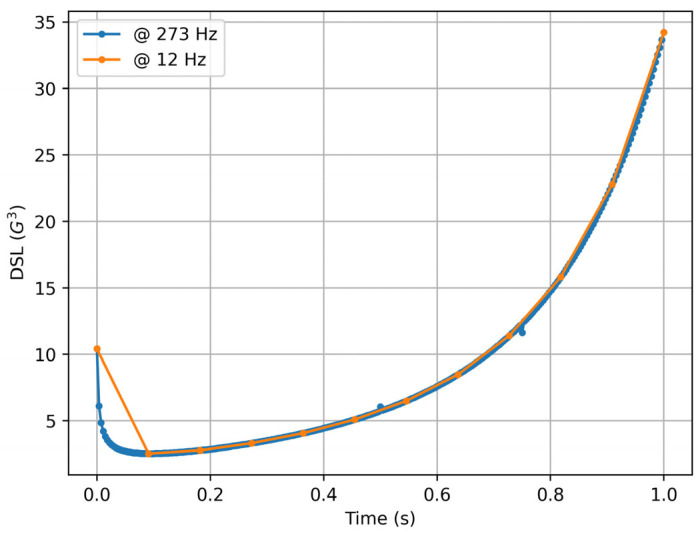
uDSL, computed using Equation (3), from artificial acceleration data with two points of disturbance (a value of 18 milliG), sampled at 273 (blue) and 12 (orange) Hz.

Therefore, the uDSL’s unit is G^3^, an unconventional but clearer alternative to arbitrary units. Additionally, this metric is independent of the sample rate used and insensitive to noise (see Figure 9).

**Figure 10:**
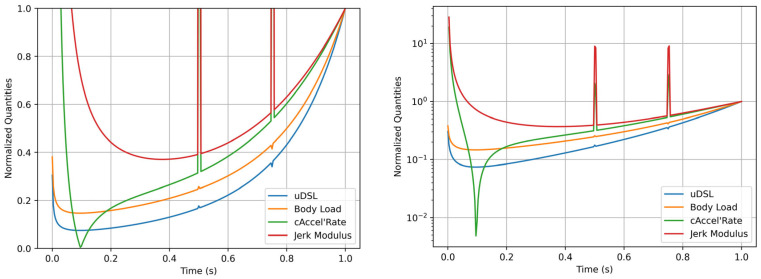
Effects of introducing noise-like data points into artificial acceleration data across each metric. On the left, the axis is normalized; on the right, the y-axis scale is logarithmic.

**Figure 11:**
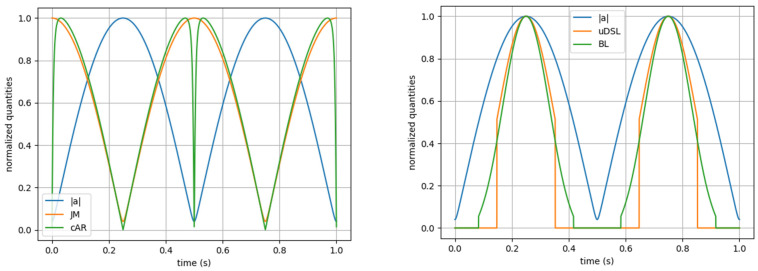
Time-series comparison between the normalized acceleration modulus (blue) and normalized mechanical load metrics. On the left, Jerk Modulus (green) and cAccel’Rate (orange). On the right, uDSL (orange) and Body Load (green).

As previously noted, there are many other acceleration-derived exertion metrics [7,23,24,25,26]. Most of these are simplified or downscaled versions of those discussed above [12], so the four selected metrics appear to be the most general and qualitatively distinct, as far as we acknowledge. They can be grouped into two categories, each comprising two almost identical metrics: derivative-based metrics, like Jerk Modulus and cAccel’Rate; and acceleration-based metrics, like uDSL and Body Load. The first group is significantly more sensitive to noise than the second group; this is illustrated in Figure 10, where all metrics are compared using artificial data with two points of disturbance (1% of the non-disturbed data). Moreover, in Figure 11 (left) it is possible to observe that derivative-based metrics contradict the acceleration modulus curve, as they are at their minimum when the acceleration modulus is at its maximum, and vice versa. In contrast, in Figure 11 (right), the acceleration-based metrics mimic the acceleration modulus curve.

In short, Table 1 displays a summary of the conceptual comparisons performed between the metrics selected for this study.

## 3. Experimental Implementation

### 3.1. Data Acquisition, Processing, and Statistical Analysis

Raw accelerometric data were collected from 33 training sessions of an elite Portuguese male handball team (Sporting Clube de Portugal) during the 2022–2023 season. The dataset comprised 463 time series (one per player per session) from 16 players (age: 26.3 ± 5.34 years) and was used to implement the revised and corrected acceleration-derived metrics. All participants and staff were fully informed and provided written consent in accordance with the Declaration of Helsinki. The research was approved by the local Ethics Committee (2023).

Players wore the sensors in an upper-back vest (near the T4–T5 vertebrae) throughout each session. The devices were calibrated and synchronized as per the manufacturer’s [27] guidelines.

The triaxial accelerometric data were collected at a native sampling rate of 100 Hz, in accordance to the common practice in accelerometry in sports sciences [28]. Since the acquisition rate was native, neither downsampling nor aliasing corrections were needed. The data were exported from the equipment manufacturer’s software into a comma-separated values (.csv) file and processed using a custom Python (version 3.11) script. No digital filters were applied, as the absence of artifacts suggested that the data had already undergone a filtering process at some stage in the acquisition chain, despite being labeled as ‘raw data’ by the company’s software.

Two correlation methods were employed. Firstly, for each subject, we computed the pairwise cross-correlation between the four metrics at zero lag using the Pearson correlation coefficient, resulting in six unique metric pairs per subject [29]. After obtaining the zero-lag Pearson cross-correlation values for each subject, we computed the mean cross-correlation across all subjects for each metric pair to quantify the overall association between metrics. Secondly, a within-subject repeated measures correlation *(r_rm_*) was performed on the accumulated forms of the various acceleration-derived metrics, with significance set at 0.05 [30].

### 3.2. Experimental Results

**Figure 12:**
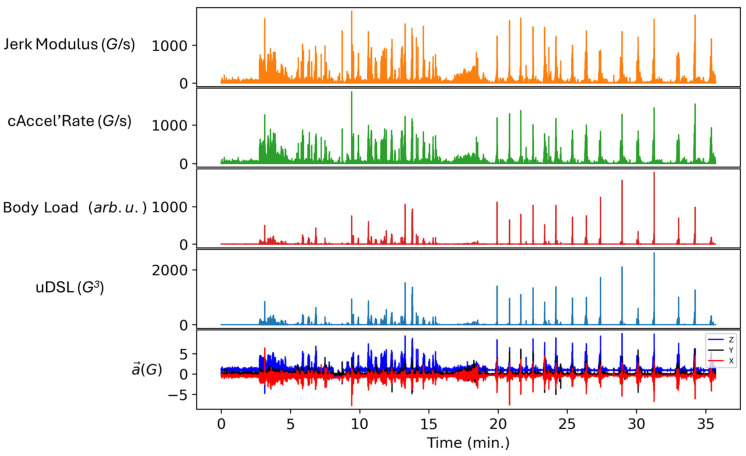
Time sequence of all metrics synchronized. From top to bottom: Jerk Modulus, cAccel’Rate, Body Load, uDSL, and original acceleration data across three axes (Z: vertical, Y: medial-lateral, X: anteroposterior).

Figure 12 provides a visual comparison of the obtained values with the revised and corrected metrics (Jerk Modulus, cAccel’Rate, Body Load, and uDSL) from a snippet of a training session of a single player. The measurement of the most intense actions—primarily jumps and landings, characterized by acceleration in the vertical axis and deceleration in the anteroposterior axis—follows a similar pattern across all metrics. Visual differences emerge in the first half of the activity, where cAccel’Rate and Jerk Modulus classify several moments as intense, whereas Body Load and uDSL categorize them as moderate or light. This discrepancy is due to the presence or absence of an intensity threshold. It is worth noting that the discrepancies present in Figure 11 are not apparent in Figure 12, due to differences in the temporal resolution between plots.

**Figure 13:**
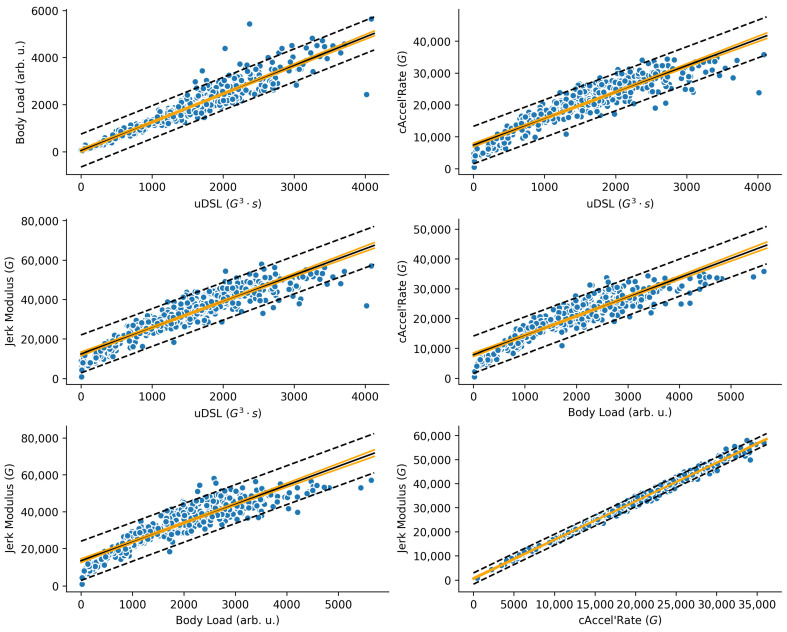
Scatterplot between every pair of metrics, with the regression line (black), the 95% confidence region (orange lines), and the 95% prediction bands (dashed lines). From top to bottom, left to right: (**top left**) uDSL (G^3^⋅s, x-axis) vs. Body Load (arb. u., y-axis); (**top right**) uDSL (G^3^⋅s, x-axis) vs. cAccel’Rate (G, y-axis); (**centre left**) uDSL (G^3^⋅s, x-axis) vs. Jerk Modulus (G, y-axis); (**centre right**) Body Load (arb. u., x-axis) vs. cAccel’Rate (G, y-axis); **(bottom left**) Body Load (arb. u., x-axis) vs. Jerk Modulus (G; y-axis); (**bottom right**) cAccel’Rate (G, x-axis) vs. Jerk Modulus (G, y-axis).

Table 2 shows descriptive statistics for each metric. Table 3 presents the mean ± SD cross-correlation coefficients for each pair of metrics. Table 4 presents repeated correlation results with confidence intervals and *p*-values, while the scatterplots (Figure 13) display regression lines, confidence regions, and prediction intervals.

## 4. Discussion

This study investigated the conceptual differences among a selection of qualitatively distinct whole-body mechanical load metrics, with the aim of improving their standardization and comparability across hardware devices. Rather than introducing new acceleration-based exertion metrics, we refined existing ones through minimal corrections that preserved their fundamentals. By implementing and comparing these metrics, we sought to better understand their applicability within the whole-body load quantification conundrum.

Using synthetic data comparisons to elucidate the conceptual differences among whole-body mechanical load metrics, minimal changes were proposed to the original equation concepts, such as considering the metrics as temporal sequences rather than solely in their summated forms, and multiplying by the sampling rate, thus ensuring hardware compatibility without altering their fundamental principles. Subsequently, the metrics were compared using experimental data to understand their statistical relationships.

Regarding derivative-based metrics such as Jerk Modulus and cAccel’Rate in this study, their independence from varying sampling rates can be achieved by simply multiplying by the inverse of the time interval, i.e., 1/Δt. In contrast, uDSL and Body Load did not require this adjustment due to their inherent independence from sampling rate variations. A proposal, aligning with previous authors [4,31], was made to rename the Player Load metric to Jerk Modulus, based on two key reasons: firstly, multiple authors have used different names for this metric, including Player Load [21], Acceleration Load [32], Body Load [33], and Total Load [23], which has led to confusion and misinterpretation; secondly, the metric actually computes the modulus of the jerk vector. The synthetic data analysis shows that, despite the corrections proposed, the architecture of these metrics can potentially amplify signal noise (Figure 3, Figure 6 and Figure 10) and, in some specific cases, produce results that contradict the acceleration values (Figure 11—left). Additionally, these metrics lack clear physical and biological meaning, which suggests that they should be avoided.

Regarding the acceleration-based metrics, the uDSL metric presents a greater challenge in terms of comprehension compared to Body Load, primarily due to its complex definition of the “impact” windows. To address this issue, a modification to the calculation of these windows was proposed, utilizing a 0.1 s moving average window. The *k* weighting factor and the threshold used in this study were selected based on previous work [18,19] with the metric, but they still lack biological validity. Among the metrics considered in this study, Body Load emerges as the most intriguing, although its origin and application remain relatively unknown. Cunniffe et al. [7] used a metric named Body Load from the same company identified by Gomez-Carmona et al. [12] as the source of the Body Load metric. However, given the fact that other metrics have been referred to by the same name despite being different [33], and that Cunniffe et al. [7] did not provide a description of the metric used, we cannot ascertain whether the metrics are, in fact, the same. Conceptually, this metric is composed of two components: a linear component, relevant for low-intensity impacts, and a cubic component, relevant for high-intensity impacts. Nevertheless, its principal conceptual limitation lies in the use of arbitrary units. From a practical standpoint, it still lacks experimental validation.

The experimental analysis produced contradictory results. The within-subject repeated measures correlation (Table 4), conducted on the accumulated form of the metrics, indicated that they are all nearly identical (r_rm_ > 0.93), suggesting interchangeability. However, the time-series cross-correlation analysis (Table 3) showed that, while cAccel’Rate and Jerk Modulus are indeed almost identical, the metrics exhibit weak to moderate mean cross-correlations with each other, with Body Load being the most distinct.

Furthermore, no clear definition of “impact” is provided or validated, and the metrics examined in this study fail to differentiate between concentric and eccentric efforts, while also neglecting the body location of the accelerometers. For instance, an accelerometer placed on the ankle is expected to show significantly higher values than one positioned on the upper back near the T4–T5 vertebrae in a landing event [34]. However, the device’s placement should not influence load quantification. Nevertheless, authors have raised concerns about the reliability of the common placement (upper back) of these devices in team sports, as it poorly represents the center of gravity [35]. To address this, metric equations should incorporate parameters that account for the placement of the accelerometer. Additionally, companies do not consistently disclose whether filtering has been applied to the data, nor the type of filtering used, before providing raw data to the sports science community [35].

## 5. Final Considerations

Researchers in sports science should take responsibility for the design and validation processes of these metrics to ensure their development is guided by methodological rigor, transparency, and innovation rather than by commercial companies offering an extensive array of variables—many of which are merely rebranded versions of existing ones with undisclosed scaling factors. Enhancing our understanding of the metrics provided by commercial systems, and how to select them, adds significant value for professionals in the field and drives companies to provide transparent filtration processes and calculation methods, and to allocate resources toward the investigation and validation of the metrics they provide.

## Figures and Tables

**Table 1:** Summary of conceptual comparison between the original acceleration-based mechanical load metrics and the applied versions.

Metric	Original Formula	Corrected Formula	Concerns
Jerk Modulus	$\sqrt{\frac{{\left({ax}_{i+1}-{ax}_{i}\right)}^{2}+{\left({ay}_{i+1}-{ay}_{i}\right)}^{2}+{\left({az}_{i+1}-{az}_{i}\right)}^{2}}{10^{2}}}$	$\frac{1}{∆t}\sqrt{{\left({ax}_{i+1}-{ax}_{i}\right)}^{2}+{\left({ay}_{i+1}-{ay}_{i}\right)}^{2}+{\left({az}_{i+1}-{az}_{i}\right)}^{2}}$	Can amplify signal noise. Contradictory results compared with acceleration norm vector.
cAccel’Rate	$\left|\sqrt{{\left(a_{x_{i}}\right)}^{2}+{\left(a_{y_{i}}\right)}^{2}+{\left(a_{z_{i}}\right)}^{2}}-\sqrt{{\left(a_{x_{i-1}}\right)}^{2}+{\left(a_{y_{i-1}}\right)}^{2}+{\left(a_{z_{i-1}}\right)}^{2}}\right|$	$\frac{1}{∆t}\left|\sqrt{{ax}_{i+1}^{2}+{ay}_{i+1}^{2}+{az}_{i+1}^{2}}-\sqrt{{ax}_{i}^{2}+{ay}_{i}^{2}+{az}_{i}^{2}}\right|$	Can amplify signal noise. Contradictory results compared with acceleration norm vector. Almost identical to Jerk Modulus.
Body Load	$bl\text{\_}{impact}_{i}=\left\{\begin{array}{c} \left\|{\vec{a}}_{i}\right\|-1⇐\left\|{\vec{a}}_{i}\right\|>1.25G\\ 0\;\;\;else \end{array}\right.$ ${BL}_{i}={bl\text{\_}impact}_{i}+{bl\text{\_}impact}_{i}^{3}$		Use of arbitrary units.
uDSL	$dsl\text{\_}{impact}_{i}=\left\{\begin{array}{c} \underset{0.1s\mathrm{window}}{\max}\left\|\vec{a}\right\|⇐\underset{0.1s\mathrm{window}}{\max}\left\|\vec{a}\right\|>2G\\ 0\;\;\;else \end{array}\right.$ $\alpha \cdot {dsl\text{\_}impact}_{i}^{k}$	${\tilde{y}}_{i}=\frac{10}{sample\;rate}\sum_{k=\left(-50\;ms\right)}^{\left(50\;ms\right)}\left|\left|{\vec{a}}_{i+k}\right|\right|$ $IMPACT_{i}=\left\{\begin{array}{c} {\tilde{y}}_{i},\;\;{\tilde{y}}_{i}>2\;G\\ 0,\;\;else \end{array}\right.$ $uDSL_{i}=IMPACT_{i}^{3}$	Opaque definition of impact windows. Opaque definition of k weighting factor.

**Table 2:** Descriptive statistics for accumulated forms of Jerk Modulus, cAccel’Rate, Body Load, and uDSL.

Metric	Mean	Min.	25th Perc.	Median	75th Perc.	Max.	SD
Jerk Modulus (G)	33,063.1	871.5	25,828.6	34,898.6	42,128.7	57,973.2	12,254.1
cAccel’Rate (G)	20,267.7	488.5	15,776.1	21,107.4	25,805.1	35,815.0	7627.4
Body Load (arb. u.)	1921.6	16.1	1172.4	1907.2	2631.3	5638.0	1076.7
uDSL (G^3^⋅s)	1549.9	5.6	930.0	1596.1	2099.3	4084.3	844.1

**Table 3:** Time-series cross-correlation coefficients (mean ± SD) between each pair of metric time series.

		r (Mean ± SD)
cAccel’Rate	Jerk Modulus	0.91 ± 0.02
	Body Load	0.28 ± 0.07
	uDSL	0.43 ± 0.08
Jerk Modulus	Body Load	0.32 ± 0.08
	uDSL	0.52 ± 0.10
Body Load	uDSL	0.42 ± 0.08

**Table 4:** Repeated measures correlation between external load metrics per training session.

		*r_rm_*	CI95%	*p*-Value
Jerk Modulus	cAccel’Rate	1	[1.00, 1.00]	<0.001
	uDSL	0.95	[0.94, 0.96]	<0.001
	Body Load	0.94	[0.93, 0.95]	<0.001
cAccel’Rate	uDSL	0.95	[0.94, 0.96]	<0.001
	Body Load	0.94	[0.93, 0.95]	<0.001
uDSL	Body Load	0.97	[0.96, 0.97]	<0.001

## Data Availability

The data utilized in this study are not available due to privacy concerns related to the team from which the data were collected.

## References

[1] Camomilla V., Bergamini E., Fantozzi S., Vannozzi G. (2018). Trends Supporting the In-Field Use of Wearable Inertial Sensors for Sport Performance Evaluation: A Systematic Review. Sensors.

[2] Staunton C.A., Abt G., Weaving D., Wundersitz D.W.T. (2022). Misuse of the term ‘load’ in sport and exercise science. J. Sci. Med. Sport.

[3] Vanrenterghem J., Nedergaard N.J., Robinson M.A., Drust B. (2017). Training Load Monitoring in Team Sports: A Novel Framework Separating Physiological and Biomechanical Load-Adaptation Pathways. Sports Med..

[4] Bredt S.D.T., Chagas M.H., Peixoto G.H., Menzel H.J., de Andrade A.G.P. (2020). Understanding Player Load: Meanings and Limitations. J. Hum. Kinet..

[5] Impellizzeri F.M., Jeffries A.C., Weisman A., Coutts A.J., McCall A., McLaren S.J., Kalkhoven J. (2022). The ‘training load’ construct: Why it is appropriate and scientific. J. Sci. Med. Sport.

[6] Trost S.G., Mciver K.L., Pate R.R. (2005). Conducting accelerometer-based activity assessments in field-based research. Med. Sci. Sports Exerc..

[7] Cunniffe B., Proctor W., Baker J.S., Davies B. (2009). An Evaluation of the Physiological Demands of Elite Rugby Union Using Global Positioning System Tracking Software. J. Strength Cond. Res..

[8] Treuth M.S., Schmitz K., Catellier D.J., McMurray R.G., Murray D.M., Almeida M.J., Going S., Norman J.E., Pate R. (2004). Defining accelerometer thresholds for activity intensities in adolescent girls. Med. Sci. Sports Exerc..

[9] Rowlands A.V., Stiles V.H. (2012). Accelerometer counts and raw acceleration output in relation to mechanical loading. J. Biomech..

[10] Delves R.I.M., Aughey R.J., Ball K., Duthie G.M. (2021). The Quantification of Acceleration Events in Elite Team Sport: A Systematic Review. Sports Med.—Open.

[11] Montgomery P.G., Pyne D.B., Minahan C.L. (2010). The Physical and Physiological Demands of Basketball Training and Competition. Int. J. Sport Physiol..

[12] Gomez-Carmona C.D., Bastida-Castillo A., Ibanez S.J., Pino-Ortega J. (2020). Accelerometry as a method for external workload monitoring in invasion team sports. A systematic review. PLoS ONE.

[13] García-Sánchez C., Navarro R.M., Karcher C., de la Rubia A. (2023). Physical Demands during Official Competitions in Elite Handball: A Systematic Review. Int. J. Environ. Res. Public Health.

[14] Gómez-Carmona C.D., Pino-Ortega J., Sánchez-Ureña B., Ibáñez S.J., Rojas-Valverde D. (2019). Accelerometry-Based External Load Indicators in Sport: Too Many Options, Same Practical Outcome?. Int. J. Environ. Res. Public Health.

[15] Gaudino P., Iaia F.M., Strudwick A.J., Hawkins R.D., Alberti G., Atkinson G., Gregson W. (2015). Factors influencing perception of effort (session rating of perceived exertion) during elite soccer training. Int. J. Sports Physiol. Perform..

[16] Hollville E., Couturier A., Guilhem G., Rabita G. (2021). A Novel Accelerometry-Based Metric to Improve Estimation of Whole-Body Mechanical Load. Sensors.

[17] Mateus N., Abade E., Coutinho D., Gómez M.-Á., Peñas C.L., Sampaio J. (2025). Empowering the Sports Scientist with Artificial Intelligence in Training, Performance, and Health Management. Sensors.

[18] Buchheit M., Laursen P.B. (2024). Sports Science 3.0: Integrating Technology and AI with Foundational Knowledge. Sport Perform. Sci. Rep..

[19] Gabbett T.J., Oetter E. (2025). From Tissue to System: What Constitutes an Appropriate Response to Loading?. Sports Med..

[20] Hanson B., Stall S., Cutcher-Gershenfeld J., Vrouwenvelder K., Wirz C., Rao Y.D., Peng G. (2023). Garbage in, garbage out: Mitigating risks and maximizing benefits of AI in research. Nature.

[21] Boyd L.J., Ball K., Aughey R.J. (2011). The Reliability of MinimaxX Accelerometers for Measuring Physical Activity in Australian Football. Int. J. Sport Physiol..

[22] Beato M., De Keijzer K.L., Carty B., Connor M. (2019). Monitoring Fatigue During Intermittent Exercise With Accelerometer-Derived Metrics. Front. Physiol..

[23] Bowen L., Gross A.S., Gimpel M., Li F.-X. (2017). Accumulated workloads and the acute: Chronic workload ratio relate to injury risk in elite youth football players. Br. J. Sports Med..

[24] Reche-Soto P., Cardona-Nieto D., Diaz-Suarez A., Bastida-Castillo A., Gomez-Carmona C., Garcia-Rubio J., Pino-Ortega J. (2019). Player Load and Metabolic Power Dynamics as Load Quantifiers in Soccer. J. Hum. Kinet..

[25] Dalen T., Jorgen I., Gertjan E., Havard H.G., Ulrik W. (2016). Player Load, Acceleration, and Deceleration during Forty-Five Competitive Matches of Elite Soccer. J. Strength Cond. Res..

[26] Colby M.J., Dawson B., Heasman J., Rogalski B., Gabbett T.J. (2014). Accelerometer and GPS-Derived Running Loads and Injury Risk in Elite Australian Footballers. J. Strength Cond. Res..

[27] WIMU PRO (2022). Hudl 9.87.

[28] Migueles J.H., Cadenas-Sanchez C., Ekelund U., Delisle Nyström C., Mora-Gonzalez J., Löf M., Labayen I., Ruiz J.R., Ortega F.B. (2017). Accelerometer Data Collection and Processing Criteria to Assess Physical Activity and Other Outcomes: A Systematic Review and Practical Considerations. Sports Med..

[29] Thomas S.J., Zeni J.A., Winter D.A. (2022). Winter’s Biomechanics and Motor Control of Human Movement.

[30] Bakdash J.Z., Marusich L.R. (2017). Repeated Measures Correlation. Front. Psychol..

[31] Staunton C., Wundersitz D., Gordon B., Kingsley M. (2020). Discrepancies Exist between Exercise Prescription and Dose in Elite Women’s Basketball Pre-Season. Sports.

[32] Saal C., Baumgart C., Wegener F., Ackermann N., Sölter F., Hoppe M.W. (2023). Physical match demands of four LIQUI-MOLY Handball-Bundesliga teams from 2019–2022: Effects of season, team, match outcome, playing position, and halftime. Front. Sports Act. Living.

[33] Aguiar M.V.D., Botelho G.M.A., Gonçalves B.S.V., Sampaio J.E. (2013). Physiological Responses and Activity Profiles of Football Small-Sided Games. J. Strength Cond. Res..

[34] Gómez-Carmona C.D., Bastida-Castillo A., González-Custodio A., Olcina G., Pino-Ortega J. (2020). Using an Inertial Device (WIMU PRO) to Quantify Neuromuscular Load in Running: Reliability, Convergent Validity, and Influence of Type of Surface and Device Location. J. Strength Cond. Res..

[35] Edwards S., White S., Humphreys S., Robergs R., O’Dwyer N. (2019). Caution using data from triaxial accelerometers housed in player tracking units during running. J. Sports Sci..

